# Population size estimation of female sex workers in Iran: Synthesis of methods and results

**DOI:** 10.1371/journal.pone.0182755

**Published:** 2017-08-10

**Authors:** Hamid Sharifi, Mohammad Karamouzian, Mohammad Reza Baneshi, Mostafa Shokoohi, AliAkbar Haghdoost, Willi McFarland, Ali Mirzazadeh

**Affiliations:** 1 HIV/STI Surveillance Research Center, and WHO Collaborating Center for HIV Surveillance, Institute for Futures Studies in Health, Kerman University of Medical Sciences, Kerman, Iran; 2 Department of Biostatistics and Epidemiology, Faculty of Public Health, Kerman University of Medical Sciences, Kerman, Iran; 3 School of Population and Public Health, Faculty of Medicine, University of British Columbia, Vancouver, BC, Canada; 4 Modeling in Health Research Center, Institute for Futures Studies in Health, Kerman University of Medical Sciences, Kerman, Iran; 5 Epidemiology and Biostatistics, Schulich School of Medicine & Dentistry, The University of Western Ontario, London, Canada; 6 Global Health Sciences, University of California, San Francisco, California, United States of America; 7 Department of Epidemiology and Biostatistics, University of California, San Francisco, California, United States of America; Hokkaido University Graduate School of Medicine, JAPAN

## Abstract

**Introduction:**

Estimating the number of key populations at risk of HIV is essential for planning, monitoring, and evaluating prevention, care, and treatment programmes. We conducted this study to estimate the number of female sex workers (FSW) in major cities of Iran.

**Methods:**

We used three population size estimation methods (i.e., wisdom of the crowds, multiplier method, and network scale-up) to calculate the number of FSW in 13 cities in Iran. The wisdom of the crowds and multiplier methods were integrated into a nationwide bio-behavioural surveillance survey in 2015, and the network scale-up method was included in a national survey of the general population in 2014. The median of the three methods was used to calculate the proportion of the adult female population who practice sex work in the 13 cities. These figures were then extrapolated to provide a national population size estimation of FSW across urban areas.

**Results:**

The population size of FSW was 91,500 (95% Uncertainty Intervals [UIs] 61,400–117,700), corresponding to 1.43% (95% UIs 0.96–1.84) of the adult (i.e., 15–49 year-old) female population living in these 13 cities. The projected numbers of FSW for all 31 provincial capital cities were 130,800 (95% UIs 87,800–168,200) and 228,700 (95% UIs 153,500–294,300) for all urban settings in Iran.

**Conclusions:**

Using methods of comparable rigor, our study provided a data-driven national estimate of the population size of FSW in urban areas of Iran. Our findings provide vital information for enhancing HIV programme planning and lay a foundation for assessing the impact of harm reduction efforts within this marginalized population.

## Introduction

Estimating the size of key populations at risk for HIV (e.g., female sex workers [FSW], men who have sex with men [MSM], and people who inject drugs [PWID]) is essential for understanding the magnitude and burden of the epidemic, developing appropriate prevention and treatment programs, gauging service coverage, and allocating resources [1]. However, the hidden and marginalized nature of these key populations create significant challenges in estimating their numbers through gold standard methods (e.g., census, household survey). These challenges are even more formidable in settings where cultural (e.g., the extremely stigmatized nature of sex work) and legal (e.g., the criminalization of sex work) proscriptions are severe [2, 3].

Although the HIV epidemic in Iran has been concentrated in PWID [4], FSW have also been affected. Nationwide integrated biological and behavioural surveillance (IBBS) surveys in Iran estimate HIV prevalence among FSW at 4.5% (95% Uncertainty Intervals [UIs] 2.4–8.3) in 2010 [4] and 2.1% (95% UIs 1.4–3.0) in 2015 [5]. The existing estimate for the number of FSW in Iran (80,000 or 0.5% of the adult female population) is based on expert opinion and anecdotal evidence of their presence in certain venues [4, 6]. A more evidence-based estimate of the absolute number of FSW in Iran is needed.

Given the absence of a single gold standard or bias-free approach, we adapted and applied several methods, namely wisdom of the crowds (WOTC) [7, 8], service and unique object multipliers [1, 3, 9], and network scale-up (NSU) [10, 11]), to estimate the population size of FSW in Iran. We synthesized the findings of these several approaches to arrive at a more robust estimate to better inform health policies and guide research to improve the health of FSW in Iran.

## Methods

### Overall study design, data sources, and setting

We used three population size estimation methods to estimate the number of FSW in Iran. The WOTC [7, 8] and multiplier methods were integrated into the IBBS [3] among FSW in 13 cities conducted in 2015 [12]. A total of 1,337 eligible FSW were recruited through facility- and outreach- based sampling methods. Eligibility criteria were: 1) female sex, 2) having exchanged sex (vaginal, anal, or oral) for money, goods, or favors with at least one male partner in the past 12 months, 3) 18 years of age or older, 4) holding Iranian citizenship and residing or working in the city of the study, and 5) providing informed consent. The NSU method was integrated into a national population-based survey conducted in 13 provinces in 2014 [11]. The survey aimed to estimate the size of populations with risky sexual and drug use practices. For the NSU study, FSW were defined as women with at least one event of exchanging sex for money, goods, or favor in the past 12 months. The NSU method has been previously used and validated in Iran for other populations at risk for HIV (e.g., PWID) [11, 13].

**Fig 1:**
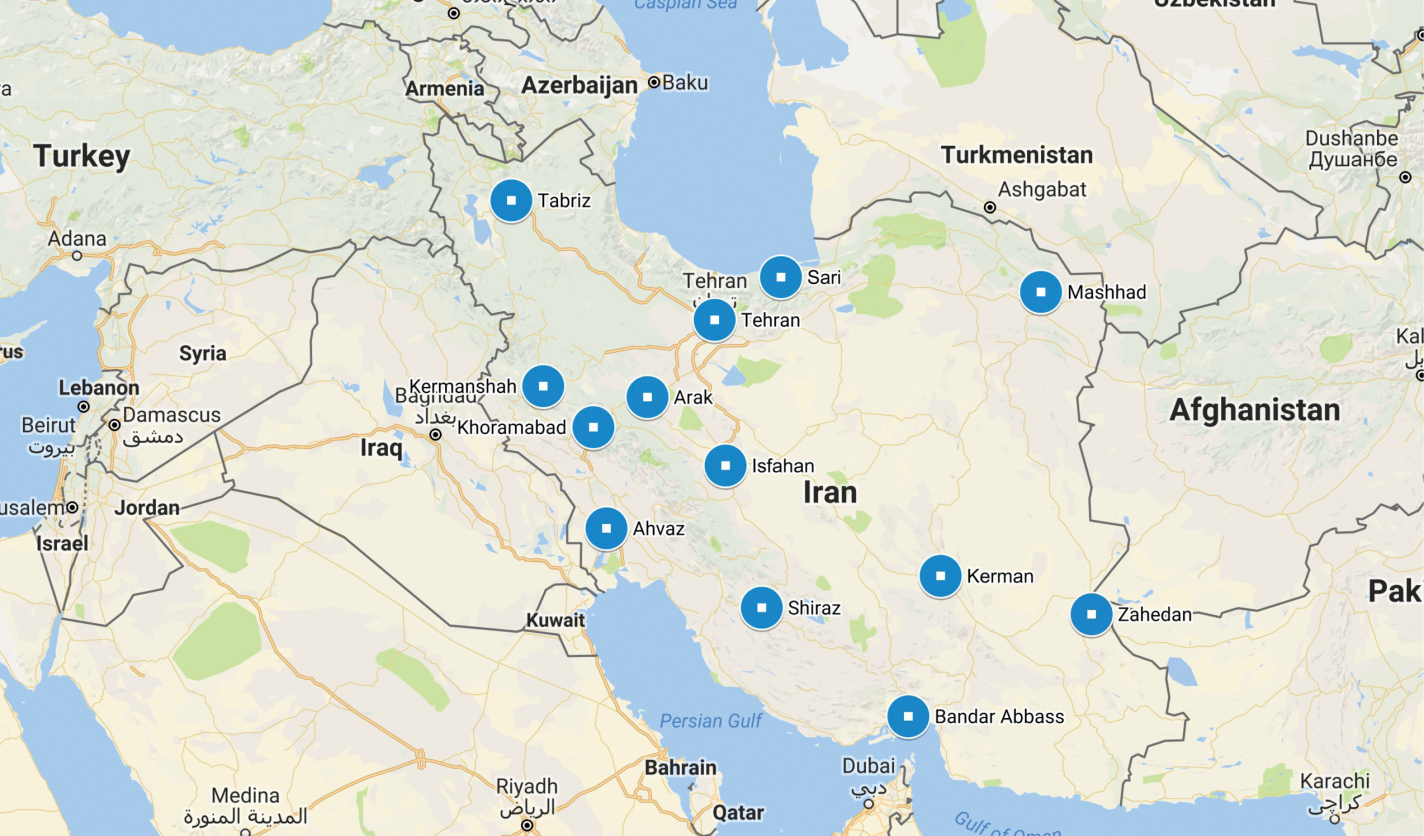
Location of study cities for the bio-behavioural survey of female sex workers, Iran, 2015. Google map used to build Fig 1. Per guidline presented in the Google website (https://www.google.com/permissions/geoguidelines.html))

The 13 geographically dispersed survey sites (Fig 1) were selected to provide a relatively representative cross section of the regions of Iran considering the required logistical support for implementing the survey and referral services (e.g., the existence of harm reduction facilities catered towards FSW). Detailed description of each size estimation methods is provided below.

### Wisdom of the crowds method

The WOTC method [7, 8] entailed asking FSW who participated in the IBBS survey to give their best guess, the lowest and highest plausible of the number of FSW in their city. The method assumes that FSW are familiar with their population, have different perspectives on their numbers, and that a sufficient number of responses can provide a reasonable approximation [14]. Response were adjusted for over- and under- estimation by two approaches. First, estimates suggesting that >3% of adult women in the city were FSW were truncated to 3% based on the upper range of size estimates in the world literature [15]. Second, estimates lower than the number of FSW recruited for the IBBS survey in the respective city were set at that number. The data were then used to calculate the median of best guess and the lowest and highest plausible number as 95% UIs for the upper and lower bound for the number of FSW in each city.

### Multiplier method

We also integrated the service and unique object multiplier methods [3, 9] into the 2015 IBBS surveys to estimate the number of FSW in the 13 cities. Estimation of the total size of the population using multiplier methods relies on two overlapping but independent sources of information. One source is the count of clients receiving a service from a facility or organisation catered towards FSW during a specified period. The present study used administrative data from social welfare organisations or facilities supported by the Ministry of Health and Medical Education (MoHME). These data included unduplicated client counts of services for HIV counseling and testing, harm reduction, addiction treatment, and clinical care. We also used participation in our previous facility-based FSW survey in 2010 as a source of the first count. The second data source was the IBBS survey, which determined the proportion of the target population who received the service at the specific facility during the specified period.

When service data are not available, the first count can be generated by distributing a unique object to members of the target population. Similar to the capture-recapture approach [1], the unique object multiplier method entails giving a “tag” (i.e., a memorable gift) shortly before the IBBS survey is implemented. For example, outreach workers distribute necklaces to FSW in the target areas before conducting the IBBS. The IBBS then measures the proportion of FSW who received the necklace. The service or object count together with the proportion of FSW who report using the service or receiving the object in the IBBS are used to calculate the total population size using the following formula [3]:

$$N=\frac{M}{P}$$ (1)

Where *N* is the estimated total population size of FSW, *P* is the proportion of those who reported receiving the necklace or the service, and *M* is the total number of necklaces distributed or count of FSW who received the service.

To approximate the 95% UIs around the population size estimation, the variance was calculated using the Delta Method [3] (see below) with the 95% UIs for N calculated as $N\pm 1.96\sqrt{Var(N)}$. The E(P) and E(M) represent the average of P and M, respectively.

$$Var(N)=\frac{Var(M)}{{\left[E(P)\right]}^{2}}+\frac{{\left[E(M)\right]}^{2}}{{\left[E(P)\right]}^{4}}Var(P)$$ (2)

### Network scale-up method

The NSU method for population size estimation is based on the assumption that the prevalence of a risky behavior within the social networks of a representative sample of the general population reflects the prevalence in the whole population. The NSU method relies on asking participants about the risky behaviors among their acquaintances in their social network, rather than asking the question of the participants themselves [10, 16]. The approach is less prone to the biases of under-reporting stigmatised behaviors.

The present study applied the NSU approach to estimate the proportion of FSW within the social network of participants in a general population survey conducted in 2014 [11]. Using a random street walk sampling design, 500 to 1,000 adults were recruited in each city totalling 6,945 participants. The trained interviewers approached adult men and women of various age and socio-demographics in public places (e.g., malls, streets, and parks) and briefed them about the aims of the study. Previous literature suggests that in the context of collecting data on sensitive topics, anonymous street-based data collection provides more reliable responses compared to household or telephone-based surveys in Iran [17]. Consenting individuals participating in face-to-face gender-matched interviews were asked about the number of FSW they knew. “Knowing” someone was defined as “people whom you know, and they know you, in appearance or by name, with whom you can interact if needed, and with whom you have contacted over the last two years personally, by phone, or via e-mail”[18]. The crude population size of FSW was calculated using the following formula [19]:

$$N=t\frac{\sum_{i}m_{i}}{\sum_{i}c_{i}}$$ (3)

Where *t* is the total adult (15–49) female population of each city, *c* is the average social network size of the participants, *m* is the number of FSW known the participants, *N* is the population size of FSW, and *i* index stands for the respondent group, the general population surveyed. Our analysis was informed by the average social network size of the general Iranian population estimated at 308 persons [18]. To adjust for the transparency barrier bias, we used the correction factor measured by Maghsoudi et al [20]. They reported only 54% of general population were aware of sex work involvement of their acquaintances (i.e., transparency barrier bias). In our analysis, we multiplied the crude estimates to 1 ÷ 54% = 1.85 (i.e., visibility factor) to adjust the crude estiamtes for transparency barrier bias. We calculated the 95% UIs using Monte-Carlo simulation, assuming a Poisson distribution (i.e., with mean equal to that of the responses) for *N* and uniform distribution (i.e., in the range of 1 ÷ 64% = 1.56 to 1 ÷ 44% = 2.27) for the visibility factor.

### Synthesis and extrapolation

We used the separate estimates in the above-mentioned methods to reach a “best estimate” for the number of FSW per city and overall. We converted the absolute numbers of FSW into percentages of the female population aged 15 to 49 years and applied the median of the percentages to each city as the “best” population size estimate. The median of the 13 cities’ size estimates was used as the overall FSW population for all urban areas of Iran. The same approach was used for the lower and upper limits to calculate the acceptability bounds for all study cities and the overall urban population. In this manner, the median, upper, and lower limit proportions of the 13 cities in the study were applied to all 31 provincial capitals and other major cities in Iran to extrapolate estimates of FSW to the national level for urban areas.

### Ethical considerations

All participants provided verbal consent after the study purpose, procedures, potential harm, and benefits were explained to them by a study staff member. A staff member signed a consent form for each study participant. A waiver of written informed consent was requested as allowed under human subjects research regulation 45CFR46.117(c). The study met both conditions in that: 1) the signed consent would have been the only record that could link respondents to the research, and 2) the research presented no more than minimal risk of harm and involved no procedures for which written consent is normally required outside of research. The ethics committee of the Kerman University of Medical Sciences, Iran reviewed and approved the study protocol and procedures of FSW IBBS (IR.KMU.REC.94.611) and the NSU general population survey (IR.KMU.90.163).

## Results

### Wisdom of the crowds estimates

Of the 1,337 FSW participants in the IBBS, 840 (62.8%) answered the WOTC question in 12 of the 13 study cities (Table 1). The overall median response for the FSW population size translated to 2.38% (95% UIs: 1.46–3.35) of 15 to 49 year-old women residing in the study cities or 152,200 (95% UIs: 93,400–214,300) in absolute numbers. The proportions ranged from a low of 0.51% in Zahedan to a high of 2.87% in Bandar-Abbas. The FSW point estimate fell between 2.02% and 2.87% for eight of the 12 cities where the WOTC method was used.

**Table 1:** Population size estimates of female sex workers in 12 of 13 study cities in Iran using the wisdom of crowds method, 2015.

City (female population age 15–49 years)	Absolute size estimate, N (uncertainty intervals)	As a percent of women age 15–49 years, % (uncertainty intervals)
Ahvaz (349,622)	10,000 (5,400–13,500)	2.86 (1.55–3.86)
Arak (165,551)	3,800 (2,600–5,600)	2.30 (1.57–3.38)
Bandar Abbas (139,274)	4,000 (2,200–6,100)	2.87 (1.58–4.45)
Isfahan (605,142)	12,200 (7,800–16,600)	2.02 (1.29–2.74)
Kerman (186,958)	4,600 (2,500–6,200)	2.46 (1.34–3.32)
Kermanshah (269,468)	1,600 (1,200–5,300)	0.59 (0.45–1.97)
Khoram Abad (113,872)*	----	----
Mashhad (841,327)	12,000 (6,700–16,900)	1.43 (0.80–2.01)
Sari (94,251)	800 (400–1,100)	0.85 (0.42–1.17)
Shiraz (484,098)	13,300 (8,700–17,800)	2.75 (1.80–3.68)
Tabriz (461,422)	13,100 (9,000–18,200)	2.84 (1.95–3.94)
Tehran (2,521,909)	63,700 (44,500–96,700)	2.52 (1.76–3.83)
Zahedan (162,927)	840 (500–2,300)	0.51 (0.31–1.41)
**Median**	**152,200 (93,400–214,300)**	**2.38 (1.46–3.35)**

*No participants in Khoram Abad answered the wisdom of the crowds question.

### Multiplier estimates

Averaging the various multipliers (see S1 Table) in each city, the overall median proportion of FSW among adult women was 0.31% (95% UIs: 0.17–0.59), corresponding to 19,800 (95% UIs: 10,900–38,100) for 12 of the 13 study cities in Iran that used the multiplier method (Table 2). The lowest estimate was 0.03% in Kermanshah, and the highest was 5.0% in Sari. Of note, the proportion fell below 1.0% for ten of the 12 cities where the multiplier method was applied.

**Table 2:** Population size estimates of female sex workers in 12 of 13 study cities in Iran using median of different multipliers methods, 2015.

City	Absolute size estimate, N (uncertainty intervals)	As a percent women age 15–49 years, % (uncertainty intervals)
Ahvaz	1,200 (180–8,500)	0.35 (0.05–2.43)
Arak	3,000 (500–21,900)	1.81 (0.28–13.20)
Bandar Abbas	390 (170–900)	0.28 (0.12–0.65)
Isfahan	2,300 (1,150–5,850)	0.38 (0.190–0.97)
Kerman	1,400 (200–9,700)	0.73 (0.11–5.17)
Kermanshah	70 (40–120)	0.03 (0.01–0.04)
Khoram Abad	200 (150–290)	0.17 (0.13–0.25)
Mashhad	3,000 (1700–5,300)	0.35 (0.20–0.63)
Sari	4,700 (1,000–6,600)	5.00 (1.06–7.00)
Shiraz	1,300 (700–22,700)	0.26 (0.13–0.54)
Tabriz	170 (50–700)	0.04 (0.01–0.15)
Tehran	7,500 (1,600; 42300)	0.30 (0.06; 1.68)
Zahedan *	----	----
**Median**	**19,800 (10,900–38,100)**	**0.31 (0.17–0.58)**

*No multiplier was done in Zahedan.

### Network scale-up estimates

Using the NSU method, the overall proportion of FSW among women in the 13 cities was 1.54% (95% UIs: 1.36–1.71), corresponding to 98,500 (95% UIs: 87,000–109,400) FSW (Table 3). FSW prevalence ranged from 0.14% (Tabriz) to 2.44% (Isfahan) of adult women. The FSW proportion was between 1.0% and 2.0% for ten of the 13 cities.

**Table 3:** Population size estimates of female sex workers in 13 study cities in Iran using the network scale-up method, 2014.

City	Absolute size estimate, N (uncertainty intervals)	As a percent of women age 15–49 years, % (uncertainty intervals)
Arak	2,200 (1,700–2,600)	1.30 (1.05–1.55)
Ahvaz	4,300 (3,300–5,200)	1.22 (0.96–1.47)
Bandar Abbas	2,200 (1,800–2,500)	1.56 (1.31–1.84)
Isfahan	14,700 (13,100–16,500)	2.44 (2.16–2.74)
Kerman	2,000 (1,500–2,500)	1.06 (0.85–1.31)
Kermanshah	4,000 (3,300–4,700)	1.47 (1.23–1.75)
Khoram Abad	740 (570–930)	0.65 (0.50–0.80)
Mashhad	15,200 (12,500–18,100)	1.81 (1.49–2.16)
Sari	1,500 (1,200–1,700)	1.54 (1.30–1.81)
Shiraz	8,100 (7,100–9,100)	1.67 (1.46–1.890)
Tabriz	640 (420–930)	0.14 (0.09–0.19)
Tehran	38,700 (34,200–43,400)	1.54 (1.36–1.71)
Zahedan	2,600 (2,200–3,000)	1.63 (1.38–1.88)
**Median**	**98,500 (87,000–109,400)**	**1.54 (1.36–1.71)**

### Synthesis and extrapolation of estimates

**Fig 2:**
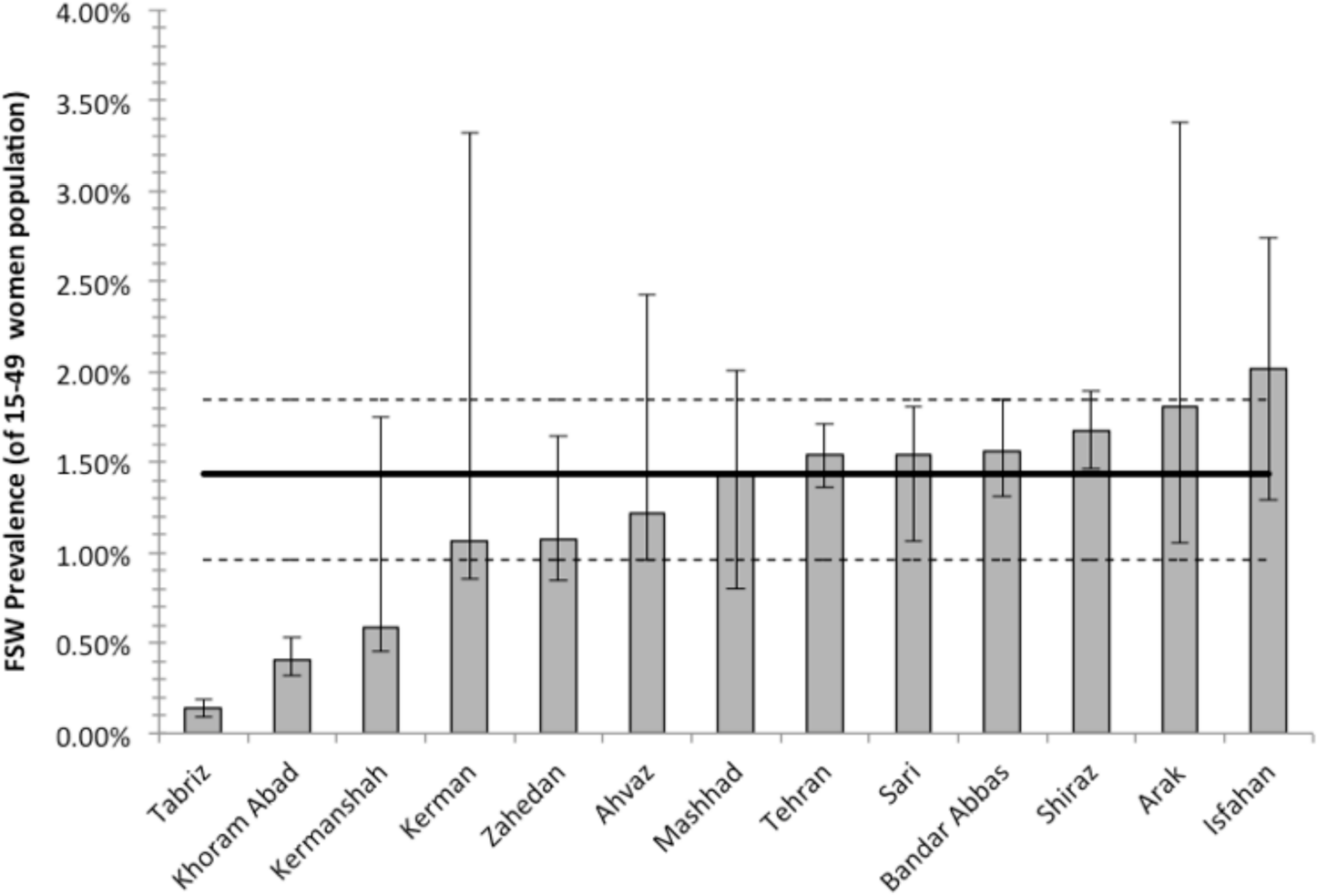
Proportion of female sex workers in the population of women 15–49 years old in 13 Iranian cities, 2015. The solid line is the final summary estimate and the dashed lines are upper and lower 95% uncertainty intervals. Error bars are 95% uncertainty intervals for each city.

We calculated the point and 95% UIs for each of the population size estimates across all the above-mentioned methods in each city. We then calculated the median of all population size estimations (separately for point, 95% uncertainity lower and upper bounds) calculated by WOTC, multiplier, and NSU methods. Fig 2 presents the final city-level estimates for the proportion of FSW among the female population aged 15 to 49 years old. The proportion ranged from 0.14% in Tabriz to 2.02% in Isfahan. Using the median of the 13 city-level estimates of 1.43% (0.96–1.84), we projected the total population size of FSW in all 13 cities to be 91,500 (95% UIs: 61,400–117,700) in 2015. Considering the total population of women aged 15–49 years in the remaining 18 provincial capital cities in Iran (i.e., 2,747,528 women), we estimated 39,300 (95% UIs: 26,400–50,500) additional FSW. Therefore, the population size of FSW in all provincial capital cities in Iran was estimated to be 130,800 (95% UIs: 87,800–168,200). Applying the same proportion to all other major cities, our best estimate for the population size of FSW in urban Iran is 228,700 (95% UIs: 153,500–294,300).

## Discussion

The synthesis of our data suggests that one in 70 urban Iranian women age 15–49 years (1.43%) had exchanged sex for money, goods, or favor in the previous year. This translates to nearly one-quarter of a million women. Given the estimated HIV prevalence of 2.1% among FSW in the 2015 national IBBS survey [5, 21], the projected number of HIV-positive FSW would be 4,803 in urban Iran. As of September 2013, Iranian case records indicate 2,047 women reported with HIV were infected through heterosexual transmission [22]. Given that most of the identified cases of women were infected through sex with their injection drug using partners, it is likely that most FSW living with HIV in Iran remain undiagnosed and outside of care and treatment.

While there is noted variability across cities and PSE methods, most estimates of FSW fell within the range of 1% to 2% of adult women. The consistency of estimates observed across the cities and methods increases the confidence in the robustness of our estimates. Our estimates are also in line with international and regional figures. For example, in their review of FSW population size estimates in different regions, Vandepitte *et al*. reported the proportion of FSW to be between 0.4% and 4.3% of the population of adult women [15]. While no data were reported from the Middle East, the proportion in Asia ranged between 0.2% and 2.6%. Our data-driven estimate for urban Iran (228,700), however, is considerably higher than the former expert-driven national population size estimate of 80,000 FSW [4], which falls in the lower end of the international range (0.5%). Of note, this expert-driven figure falls below our lower uncertainty interval (153,500). Moreover, a recent paper on the population size of FSW in Tehran that used a direct capture-recapture method [23], estimated a total of 690 (633 to 747) FSW in 2016. Their estimate looks to be more generalizable to a few neighborhoods in the south of Tehran, not the whole city, and also likely to be very underestimated given the dependency between the capture and recapture rounds. While the conservative context of Iran may reduce the visibility of sex work in the country, our findings are comparable with the narrow body of evidence on FSW size estimation studies in similar contexts. For example, a data synthesis of several PSE methods (i.e., literature review, unique object multiplier, capture-recapture, WOTC, and service multiplier methods) in Unguja Island, Zanzibar (i.e., a predominantly Muslim setting, but also a major tourist destination) suggests 1.6% of adult women on the island had engaged in sex work [24].

With one exception, the WOTC provided the highest estimates across all three PSE methods in our study. This finding should be interpreted with caution given the subjectiveness of the method and its succeptibility to several biases and unmeasured influences which may lead to under- or over- estimations. Indeed, previous studies applying multiple PSE methods including WOTC, the WOTC method have shown both under-estimation (e.g., MSM in Nairobi in Kenya, FSW in Yangon and Mandalay in Myanmar [14, 25]), or over-estimation (e.g., MSM in Georgia [9]) of the population size in compare with other size estimation methods. Considering the highly criminalized and stigmatised nature of sex work in Iran [6], FSW might have exaggerated the estimates in an effort to normalize their sex work practice. In this scenario, the more uncommon sex work is in an area, the more FSW may overstate the issue. The fact that WOTC-driven estimates were negatively (but non-significantly) correlated with the estimates obtained from the multiplier and NSU methods may provide this hypothesis with some support. On the other hand, feelings of isolation might result in under-estimation using the WOTC method. Indeed, the major assumption of the WOTC method is that for the respondents to know the true numbers of FSW in their town which is challenging to verify. The low WOTC estimates in Sari might be explained by a small and isolated network of FSW in this city, however, we were unable to find any study about FSW and their network size in Sari. Overall, the direction of the bias for WOTC is unknown and needs further assessment in future studies. Lastly, considering the challenges of implementing the WOTC method, future studies applying the method would benefit from cognitive testing of the question, or breaking down the city population into more “knowable” segments as done in Yangon, Myanmar [25].

Conversely, the multiplier method yielded the lowest estimates. This could be due to the lack of independence between the two data sources informing the estimation (i.e., people contacted by a certain service may be more likely to have participated in the survey) that would result in underestimating the population size [3, 26]. Indeed, we recruited participants for the current IBBS from the same facilities where the previous 2010 round. Similar positive correlations could be present for counts from the social welfare organisation services, HIV testing, and the distribution of the unique objects.

The NSU results were the closest to the median of the three methods, although this was not necessarily true for all city-level estimates. Similar to other PSE methods, NSU is prone to a number of bases including imperfect knowledge of the behaviors of persons in one’s social network and the influences of stigma on the visibility of certain behaviors [20]. While our findings might imply that the NSU method yields the most unbiased estimates with the least variability compared to the other two methods, we acknowledge that a gold standard PSE method is non-existent and therefore the NSU findings should also be interpreted with caution.

In addition to the potential biases of each method mentioned above, we acknowledge other limitations of our study. Our number were projected for women age 15–49 years, however, an unknown but important proportion of FSW might be younger than 15 or older than 49. Indeed, the last two national IBBS surveys in 2010 and 2015 suggest that just under 4% of FSW were 50 years or older. While we assume that most sex work occurs in the larger cities, our findings are also of limited generalisability to smaller cities or rural areas in Iran. While the presence of “hotspots” of sex work in particular non-urban areas is plausible, data to confirm or refute this assumption are unavialable. As no single method can be definitively shown to be a gold standard or superior to other methods, we believe our current estimate to be more data-driven than prior attempts [4] and a good first step in improving our understanding of the population size of FSW in Iran.

## Conclusions

Our national and city-level estimates of FSW population based on multiple methods provided a more evidenced-based and robust estimation of the total number of FSW in Iran. This population denominator can inform modeling exercises aimed at estimating the number of women living with HIV among this marginalized population. Our findings can be used to advocate for and mobilize resources to prevent from further transmission of HIV among FSW and their network of clients and other partners.While acknowledging uncertainties, our estimates of the number of female sex workers in Iranian cities provide a foundation for planning specialised HIV services. At a minimum, our findings help set targets for gauging progress towards the UNAIDS 90-90-90 goals (i.e., having 90% of HIV-positive FSW diagnosed, 90% of the identified cases on antiretroviral treatment, and 90% of those receiving treatment achieve viral suppression) [27]. Without a clearer sense of the number of FSW, progress in the response to the epidemic may be masked by a general population estimate towards achieving the UNAIDS goals. In fact, our population size estimates, combined with prevalence data from IBBS surveys, indicate that current approaches are falling far short of meeting these goals for FSW. Our population size estimates provide realistic targets for macro- and micro- level planning of HIV prevention and care delivery programmes in the major cities of Iran. As the first population size estimation of FSW in the Middle East, our findings highlight the feasibility of the approaches in similar settings and should be considered an integral part of national HIV prevention programmes for this population throughout the region.

## Supporting information

S1 Table The population size of female sex workers in 13 cities in Iran using different multiplier method, 2015. (DOCX)

S1 File NSU Questionnaire Farsi. (DOCX)

S2 File NSU Questionnaire English. (DOCX)

S3 File WOTC and Multiplier Questionnaire Farsi. (DOC)

S4 File WOTC and Multiplier Questionnaire English. (DOC)
