# Supplementary material for: Population size estimation of female sex workers in Iran: Synthesis of methods and results
Source: PLoS One. 2017 Aug 10;12(8):e0182755. doi: 10.1371/journal.pone.0182755 (PMC5552099; doi:10.1371/journal.pone.0182755)
Supplement: S1 Table — (DOCX) [file pone.0182755.s001.docx]

**S1 Table.** The population size of female sex workers in 13 cities in Iran using different multiplier method, 2015

| City | Method | Crude size estimate women (15-49) population (95% UIs^*^) | Size percent adult women (15-49) population (95% UIs) |
| --- | --- | --- | --- |
| Shiraz | Contribution to the similar study in 2010 | 1,900 (900; 4,200) | 0.39 (0.18; 860) |
|  | Unique Object Multiplier | 600 (400; 1,100) | 0.13 (0.08; 0.22) |
|  | **Median estimate of the multiplier** | **1,300 (700; 22,700)** | **0.26 (0.13; 0.54)** |
| Mashhad | Contribution to the similar study in 2010 | 1,600 (1,000; 2,600) | 0.19 (0.12; 0.31) |
|  | Unique Object Multiplier | 420 (260; 720) | 0.05 (0.03; 0.09) |
|  | Refer to women’s addiction treatment centre (AkbarAbad campus) in the current year | 3,000 (1,700; 5,300) | 0.35 (0.20; 0.63) |
|  | Refer to the shelter (Noodeh) in the current year | 2,900 (800; 11,800) | 0.35 (0.09; 1.40) |
|  | Refer to the quarantine of welfare office in the current year | 20,400 (3,000; 143,100) | 2.43 (0.36; 17.01) |
|  | Refer to the transit centre (Imam Ali) in the current year | 76,900 (25,500; 241,400) | 9.14 (3.03; 28.69) |
|  | **Median estimate of the multiplier** | **3,000 (1700; 5,300)** | **0.35(0.20; 0.63)** |
| Isfahan | Contribution to the similar study in 2010 | 350 (250; 510) | 0.05 (0.04; 0.08) |
|  | Unique Object Multiplier | 500 (300; 700) | 0.08 (0.05; 0.11) |
|  | Receiving education and addiction treatment in the current year (Ayandeh Rooshan Centre) | 4,100 (2,000; 11,000) | 0.68 (0.33; 1.82) |
|  | Receiving education and addiction treatment in the current year (Mojeze zendegi Centre) | 5,100 (1,500; 43,600) | 0.84 (0.25; 7.20) |
|  | **Median estimate of the multiplier** | **2,300 (1,150; 5,850)** | **0.38 (0.190; 0.97)** |
| Tehran | Contribution to the similar study in 2010 | 4,900 (2,700; 9,000) | 0.19 (0.11; 0.36) |
|  | Unique Object Multiplier | 8,500 (1,400; 58,900) | 0.34 (0.05; 2.33) |
|  | Receiving counseling and testing (ValiAsr centre) | 6450 (1720; 25820) | 0.26 (0.07; 1.02) |
|  | Receiving Service ( Ahang Rahaee Shargh centre) | 13,300 (2,000; 92,900) | 0.53 (0.08; 3.68) |
|  | **Median estimate of the multiplier** | **7,500 (1,600; 42300)** | **0.30 (0.06; 1.68)** |
| Khoramabad | Contribution to the similar study in 2010 | 440 (320; 630) | 0.39 (0.28; 0.55) |
|  | Unique Object Multiplier | 170 (120; 260) | 0.15 (0.11; 0.23) |
|  | Receiving Harm reduction service in the (Tarannom Baran) harm reduction centre | 200 (150; 290) | 0.17 (0.13; 0.25) |
|  | **Median estimate of the multiplier** | **200 (150; 290)** | **0.17 (0.13; 0.25)** |
| Sari | Contribution to the similar study in 2010 | 4,700 (1,000; 6,600) | 5.00 (1.06; 7.00) |
| Tabriz | Contribution to the similar study in 2010 | 170 (50; 700) | 0.04 (0.01; 0.15) |
| Bandar Abbas | Contribution to the similar study in 2010 | 390 (170; 900) | 0.28 (0.12; 0.65) |
| Kerman | Contribution to the similar study in 2010 | 1,400 (200; 9,700) | 0.73 (0.11; 5.17) |
| Ahvaz | Contribution to the similar study in 2010 | 1,200 (180; 8,500) | 0.35 (0.05; 2.43) |
| Kermanshah | Contribution to the similar study in 2010 | 70 (40; 120) | 0.03 (0.01; 0.04) |
| Arak | Contribution to the similar study in 2010 | 3,000 (500; 21,900) | 1.81 (0.28; 13.20) |
| ^*^Uncertainty Intervals | | | |
