# Supplementary material for: Population size estimation of female sex workers in Iran: Synthesis of methods and results
Source: PLoS One. 2017 Aug 10;12(8):e0182755. doi: 10.1371/journal.pone.0182755 (PMC5552099; doi:10.1371/journal.pone.0182755)
Supplement: S1 File — (DOCX) [file pone.0182755.s002.docx]

مشخصات پرسشگر

نام پرسشگر:

محل پرسش : استان: شهرستان:

جنس: مرد زن

شغل پرسشگر:

تاریخ و زمان آغاز مصاحبه:

لطفا توجه فرماييد:

- **
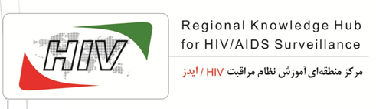
**منظور از شناختن در اين طرح "تعداد افرادی است که حداقل در دو سال گذشته يکبار با وي تماس تلفني، حضوري و يا ايميلي داشته و او را به اسم و چهره مي­شناسيد و او نيز متقابلاً شما را مي شناسد و هر زمان که لازم باشد مي توانيد به صورت تلفني، حضوري و يا ايميلي با ايشان تماس حاصل فرماييد".
- افراد پرسش شونده باید حتماً مقیم آن استان بوده و حداقل، پنج سال اخیر در آن استان زندگی کرده باشند.
- لطفا در تمامی مربع های جلوی سوالات، عدد (تعداد افراد) ذکر شود و از زدن تیک خودداری نمایید.

| **ماده مصرفی** | | |
| --- | --- | --- |
| آیا کسی را می شناسید که در یک سال گذشته حداقل یک بار یکی از **مواد مخدر، محرک یا توهم زا، چه صنعتی و چه سنتی را چه به صورت خوراکی و چه به صورت تزریقی** مصرف کرده باشد؟ | **مرد** | **زیر 18 سال 30-18 سال 50-31 سال بالای 50 سال** |
|  | **زن** | **زیر 18 سال 30-18 سال 50-31 سال بالای 50 سال** |
| از **میان افراد فوق**، چند نفر به طور مستمر و روزانه حداقل یکبار یکی از مواد مخدر، محرک یا توهم زا، چه صنعتی و چه سنتی را چه به صورت تزریقی و چه به صورت خوراکی مصرف می­کرده­اند؟ | **مرد** | **زیر 18 سال 30-18 سال 50-31 سال بالای 50 سال** |
|  | **زن** | **زیر 18 سال 30-18 سال 50-31 سال بالای 50 سال** |
| **مصرف کننده مشروبات الکلي**  چند نفر را می شناسید که در یک سال گذشته، حداقل يکبار **مشروبات الکلي (مثل عرق، شراب، آبجو، ودکا، ويسکي، الکل طبی** ...) مصرف کرده باشد؟ چه براي تفنن و چه به دليل اعتياد به آن | **مرد** | **زیر 18 سال 30-18 سال 50-31 سال بالای 50 سال** |
|  | **زن** | **زیر 18 سال 30-18 سال 50-31 سال بالای 50 سال** |
| از **میان افراد فوق**، چند نفر به طور مستمر و روزانه مشروبات الکلي (مثل عرق، شراب، آبجو، ودکا، ويسکي، الکل طبی ...) مصرف می­کرده­اند؟ | **مرد** | **زیر 18 سال 30-18 سال 50-31 سال بالای 50 سال** |
|  | **زن** | **زیر 18 سال 30-18 سال 50-31 سال بالای 50 سال** |
| **مصرف کننده تریاک، شیره یا سوخته**  چند نفر را می شناسید که در یک سال گذشته، حداقل يکبار **ترياک، شیره یا سوخته** را چه به صورت خوردنی و چه به صورت تزریقی مصرف کرده باشد؟ چه براي تفنن و چه به دليل اعتياد به آن | **مرد** | **زیر 18 سال 30-18 سال 50-31 سال بالای 50 سال** |
|  | **زن** | **زیر 18 سال 30-18 سال 50-31 سال بالای 50 سال** |
| از **میان افراد فوق**، چند نفر به طور مستمر و روزانه ترياک، شیره یا سوخته را چه به صورت خوردنی و چه به صورت تزریقی مصرف می­کرده­اند**؟** | **مرد** | **زیر 18 سال 30-18 سال 50-31 سال بالای 50 سال** |
|  | **زن** | **زیر 18 سال 30-18 سال 50-31 سال بالای 50 سال** |
| **مصرف کننده حشیش**  چند نفر را می شناسید که در یک سال گذشته، حداقل یک بار **حشیش، بنگ، چرس، سیگاری، جوینت، علف یا گراس**... مصرف کرده باشد؟ چه براي تفنن و چه به دليل اعتياد به آن | **مرد** | **زیر 18 سال 30-18 سال 50-31 سال بالای 50 سال** |
|  | **زن** | **زیر 18 سال 30-18 سال 50-31 سال بالای 50 سال** |
| از **میان افراد فوق،** چند نفر به طور مستمر و روزانه حشیش، بنگ، چرس، سیگاری، جوینت، علف یا.گراس... مصرف می­کرده­اند؟ | **مرد** | **زیر 18 سال 30-18 سال 50-31 سال بالای 50 سال** |
|  | **زن** | **زیر 18 سال 30-18 سال 50-31 سال بالای 50 سال** |
| **مصرف کننده هروئين یا کراک**  چند نفر را می شناسید که در یک سال گذشته، حداقل يکبار **هروئين یا کراک** مصرف کرده باشد؟ چه براي تفنن و چه به دليل اعتياد به آن | **مرد** | **زیر 18 سال 30-18 سال 50-31 سال بالای 50 سال** |
|  | **زن** | **زیر 18 سال 30-18 سال 50-31 سال بالای 50 سال** |
| از **میان افراد فوق،** چند نفر به طور مستمر و روزانه هروئين یا کراک مصرف می­کرده­اند | **مرد** | **زیر 18 سال 30-18 سال 50-31 سال بالای 50 سال** |
|  | **زن** | **زیر 18 سال 30-18 سال 50-31 سال بالای 50 سال** |
| **مصرف کننده مواد محرک**  چند نفر را می شناسید که در یک سال گذشته، حداقل یک بار **مت­آمفتامین، شیشه، قرص اکستازی (قرص اکس)، کوکائین یا کوک، ریتالین یا متیل فنیدیت** را بدون تجویز پزشک مصرف کرده باشد؟ چه براي تفنن و چه به دليل اعتياد به آن | **مرد** | **زیر 18 سال 30-18 سال 50-31 سال بالای 50 سال** |
|  | **زن** | **زیر 18 سال 30-18 سال 50-31 سال بالای 50 سال** |
| از **میان افراد فوق،** چند نفر به طور مستمر و روزانه مت­آمفتامین، شیشه، قرص اکستازی (قرص اکس)، کوکائین یا کوک، ریتالین یا متیل فنیدیت را بدون تجویز پزشک مصرف می­کرده­اند؟ | **مرد** | **زیر 18 سال 30-18 سال 50-31 سال بالای 50 سال** |
|  | **زن** | **زیر 18 سال 30-18 سال 50-31 سال بالای 50 سال** |
| **مصرف کننده ترامادول، دیفنوکسیلات یا ترکیبات کدئین­ دار**  چند نفر را می شناسید که در یک سال گذشته، **داروهای دارای ترکیبات افیونی همچون ترامادول، دیفنوکسیلات و استامینوفن کدئین** را مصرف کرده باشد؟ چه براي تفنن و چه به دليل اعتياد به آن | **مرد** | **زیر 18 سال 30-18 سال 50-31 سال بالای 50 سال** |
|  | **زن** | **زیر 18 سال 30-18 سال 50-31 سال بالای 50 سال** |
| از **میان افراد فوق،** چند نفر به طور مستمر و روزانه داروهای دارای ترکیبات افیونی همچون ترامادول، دیفنوکسیلات و استامینوفن کدئین مصرف می­کرده­اند؟ | **مرد** | **زیر 18 سال 30-18 سال 50-31 سال بالای 50 سال** |
|  | **زن** | **زیر 18 سال 30-18 سال 50-31 سال بالای 50 سال** |
| **مصرف کننده داروهای آرام­بخش و خواب­آور**  چند نفر را می شناسید که در یک سال گذشته، **داروهایی مثل دیازپام، آلپرازولام، کلونازپام، فنوباربیتال و**... را مصرف کرده باشد**؟** چه براي تفنن و چه به دليل اعتياد به آن | **مرد** | **زیر 18 سال 30-18 سال 50-31 سال بالای 50 سال** |
|  | **زن** | **زیر 18 سال 30-18 سال 50-31 سال بالای 50 سال** |
| از **میان افراد فوق،** چند نفر به طور مستمر و روزانه داروهایی مثل دیازپام، آلپرازولام، کلونازپام، فنوباربیتال و... مصرف می­کرده­اند؟ | **مرد** | **زیر 18 سال 30-18 سال 50-31 سال بالای 50 سال** |
|  | **زن** | **زیر 18 سال 30-18 سال 50-31 سال بالای 50 سال** |
| **مصرف کننده مواد توهم زا**  چند نفر را می شناسید که در یک سال گذشته، **موادی همچون ال اس دی، کتامین و**... را مصرف کرده باشد؟ چه براي تفنن و چه به دليل اعتياد به آن | **مرد** | **زیر 18 سال 30-18 سال 50-31 سال بالای 50 سال** |
|  | **زن** | **زیر 18 سال 30-18 سال 50-31 سال بالای 50 سال** |
| از **میان افراد فوق،** چند نفر به طور مستمر و روزانه موادی همچون ال اس دی، کتامین و... مصرف می کرده­اند؟ | **مرد** | **زیر 18 سال 30-18 سال 50-31 سال بالای 50 سال** |
|  | **زن** | **زیر 18 سال 30-18 سال 50-31 سال بالای 50 سال** |
| **مصرف کننده مواد استنشاقی**  چند نفر را می شناسید که در یک سال گذشته، موادی همچون چسب، تینر، بنزین، نیتروس اُکساید و... را مصرف کرده باشد؟ چه براي تفنن و چه به دليل اعتياد به آن | **مرد** | **زیر 18 سال 30-18 سال 50-31 سال بالای 50 سال** |
|  | **زن** | **زیر 18 سال 30-18 سال 50-31 سال بالای 50 سال** |
| از **میان افراد فوق،** چند نفر به طور مستمر و روزانه موادی همچون **چسب، تینر، بنزین، نیتروس اُکساید** و... مصرف می کرده­اند؟ | **مرد** | **زیر 18 سال 30-18 سال 50-31 سال بالای 50 سال** |
|  | **زن** | **زیر 18 سال 30-18 سال 50-31 سال بالای 50 سال** |
| **مصرف کننده مواد به روش تزريق**  چند نفر را می شناسید که در یک سال گذشته، حداقل يکبار یکی از **مواد مخدر را به** **روش تزريقي** مصرف کرده باشد**؟** | **مرد** | **زیر 18 سال 30-18 سال 50-31 سال بالای 50 سال** |
|  | **زن** | **زیر 18 سال 30-18 سال 50-31 سال بالای 50 سال** |
| از **میان افراد فوق،** چند نفر به طور مستمر و روزانه یکی از مواد مخدر را به روش تزريقي مصرف می کرده­اند**؟** | **مرد** | **زیر 18 سال 30-18 سال 50-31 سال بالای 50 سال** |
|  | **زن** | **زیر 18 سال 30-18 سال 50-31 سال بالای 50 سال** |
| **تحت درمان با متادون به صورت قانونی**  چند نفر را می شناسید که در یک سال گذشته، اعتياد به مواد مخدر داشته و تحت درمان با **متادون به صورت قانونی** قرار گرفته باشد؟ | **مرد** | **زیر 18 سال 30-18 سال 50-31 سال بالای 50 سال** |
|  | **زن** | **زیر 18 سال 30-18 سال 50-31 سال بالای 50 سال** |
| از **میان افراد فوق،** چند نفر اعتياد به مواد مخدر داشته و به طور مستمر و روزانه تحت درمان با متاون به صورت قانونی قرار گرفته است؟ | **مرد** | **زیر 18 سال 30-18 سال 50-31 سال بالای 50 سال** |
|  | **زن** | **زیر 18 سال 30-18 سال 50-31 سال بالای 50 سال** |
| **مصرف متادون به صورت غیر قانونی**  چند نفر را می شناسید که در یک سال گذشته، اعتياد به مواد مخدر داشته اما خودش **بدون تجویز پزشک و از بازار آزاد متادون** تهیه و مصرف کرده است**؟** | **مرد** | **زیر 18 سال 30-18 سال 50-31 سال بالای 50 سال** |
|  | **زن** | **زیر 18 سال 30-18 سال 50-31 سال بالای 50 سال** |
| از **میان افراد فوق،** چند نفر به طور مستمر و روزانه، خودشان بدون تجویز پزشک و از بازار آزاد متادون تهیه و مصرف می کرده­اند**؟** | **مرد** | **زیر 18 سال 30-18 سال 50-31 سال بالای 50 سال** |
|  | **زن** | **زیر 18 سال 30-18 سال 50-31 سال بالای 50 سال** |
| **مصرف کننده محصولات تنباکو**  آیا کسی را می شناسید که در یک سال گذشته، حداقل یک بار **سیگار، قلیان، پیپ، ناس** و... مصرف کرده باشد؟ چه براي تفنن و چه به دليل اعتياد به آن | **مرد** | **زیر 18 سال 30-18 سال 50-31 سال بالای 50 سال** |
|  | **زن** | **زیر 18 سال 30-18 سال 50-31 سال بالای 50 سال** |
| از **میان افراد فوق**، چند نفر به طور مستمر و روزانه سیگار، قلیان، پیپ، ناس و... مصرف می­کرده­اند؟ | **مرد** | **زیر 18 سال 30-18 سال 50-31 سال بالای 50 سال** |
|  | **زن** | **زیر 18 سال 30-18 سال 50-31 سال بالای 50 سال** |

**سوالات مربوط به ارتباطات جنسی و مسایل مربوط به آن**

| **مرداني که با زنان تن فروش تماس جنسي داشته باشند**  چند مرد را می شناسید که در یک سال گذشته، حداقل با يک زن **در ازاي پرداخت پول و يا تقبل هر گونه هزينه ديگر مالي و يا غير مالي (ارائه خدمت)** تماس جنسي داشته اند؟ | **مرد** | **زیر 18 سال 30-18 سال 50-31 سال بالای 50 سال** |
| --- | --- | --- |
| **مرداني که خارج از چارچوب خانواده با جنس مخالف تماس جنسي داشته باشند**  چند مرد را می شناسید که در یک سال گذشته، حداقل با يک زن در خارج از روابط خانوادگي تماس جنسی داشته اند؟ (**بدون پرداخت** **پول و يا تقبل هر گونه هزينه ديگر مالي و يا غير مالي (ارائه خدمت)).** | **مرد** | **زیر 18 سال 30-18 سال 50-31 سال بالای 50 سال** |
| **زنانی که خارج از چارچوب خانواده تماس جنسي با جنس مخالف داشته باشند**  چند زن را می شناسید که در یک سال گذشته، حداقل با يک مرد در خارج از روابط خانوادگي تماس جنسی داشته اند؟ (**بدون دریافت پول یا هرگونه خدمتی)** | **زن** | **زیر 18 سال 30-18 سال 50-31 سال بالای 50 سال** |
| **زنان تن­فروش**  چند زن را می شناسید که در یک سال گذشته، **در ازاي دريافت پول یا هرگونه خدمتی (مانند تأمین غذا یا محل خواب)** با افراد ديگر تماس جنسي داشته اند؟ | **زن** | **زیر 18 سال 30-18 سال 50-31 سال بالای 50 سال** |
| **چه تعداد از زنان تن فروش که در سوال فوق می شناسید، در کنار تن فروشی یک شغل درآمد زای دیگر نیز دارند؟** | | |
| **مردانی که با مردان دیگر رابطه جنسی دارند**  چند مرد را می شناسید که در یک سال گذشته، **چه در ازاي رد و بدل نمودن پول و چه براي تفنن** حداقل يکبار با يک **مرد** ديگر تماس جنسي داشته­اند؟ | **مرد** | **زیر 18 سال 30-18 سال 50-31 سال بالای 50 سال** |
| **زنی که در سال گذشته سقط جنين کرده باشد**  چند نفر زن را می شناسید که در يک سال گذشته، **سقط جنين** کرده باشند؟ | **سقط به دلیل پزشکی** | **زیر 18 سال 30-18 سال 50-31 سال بالای 50 سال** |
|  | **سقط به دلیل غیر پزشکی** | **زیر 18 سال 30-18 سال 50-31 سال بالای 50 سال** |
| **سفر به خارج از کشور برای مسایل جنسی**  چند نفر را می شناسید که در یک سال گذشته، حداقل یکبار برای **مسایل جنسی به خارج از کشور مسافرت** کرده باشند؟ | **مرد** | **زیر 18 سال 30-18 سال 50-31 سال بالای 50 سال** |
|  | **زن** | **زیر 18 سال 30-18 سال 50-31 سال بالای 50 سال** |
| **دیدن فیلم های سکسی**  چند نفر را می شناسید که در یک سال گذشته، به طور **مداوم** **فیلم های سکسی تماشا** می کرده اند؟ | **مرد** | **زیر 18 سال 30-18 سال 50-31 سال بالای 50 سال** |
|  | **زن** | **زیر 18 سال 30-18 سال 50-31 سال بالای 50 سال** |

**لطفاً جهت تحلیل بهتر نتایج به سوالات زیر، پاسخ دهید:**

**سن: شغل:**

**جنس: مرد زن**

**تحصیلات: بی­سواد/خواندن و نوشتن سیکل دیپلم دیپلم تا لیسانس بالاتر از لیسانس**

**وضعیت تاهل: مجرد متاهل مطلقه/همسر فوت کرده**

**زماان پایان مصاحبه**
