# Supplementary material for: Population size estimation of female sex workers in Iran: Synthesis of methods and results
Source: PLoS One. 2017 Aug 10;12(8):e0182755. doi: 10.1371/journal.pone.0182755 (PMC5552099; doi:10.1371/journal.pone.0182755)
Supplement: S2 File — (DOCX) [file pone.0182755.s003.docx]

NSU Questionnaire:

This questionnaire had several parts to estimate the number of different hidden groups of

| How many women do you know in last year for money or any other service (food or bed) have a sexual contact with men | Women  ………….. | <18  ……….. | 18-30  ………. | 31-50  ………… | >50  ……….. |
| --- | --- | --- | --- | --- | --- |
| - How many of them have another source of income? | …………………………. | | | | |
