# Supplementary material for: Population size estimation of female sex workers in Iran: Synthesis of methods and results
Source: PLoS One. 2017 Aug 10;12(8):e0182755. doi: 10.1371/journal.pone.0182755 (PMC5552099; doi:10.1371/journal.pone.0182755)
Supplement: S3 File — (DOC) [file pone.0182755.s004.doc]

# بخش سیزدهم: تخمین اندازه جمعیت

| شماره | سوال | پاسخ | ارجاع |
| --- | --- | --- | --- |
| 1301 | به نظر شما، چندنفر خانم در اين شهر هستندکه درازای دریافت پول، مواد مخدر و يا هر چيز و يا هر سرويس ديگری (شامل غذا، شارژ تلفن، محلی برای زندگی، بليط مسافرت و غيره)بامردان رابطه جنسی برقرارمیکنند؟ | **در کل چه تعدادی را حدس می زنيد .......................**  **کمترين تعدادی که حدس می زنيد ...........................**  **بيشترين تعدادی که حدس می زنيد ..........................**  88. نمی دانم/يادم نيست  99. بدون پاسخ | **0 نفر 🡨 س 1303** |
| 1302 | چند نفر از خانمهايی که در بالا ذکر کرديد، بالای 18 سال هستند؟اگر درصد آنرا ذکر کرد، تعداد را حساب کنيد و در اين قسمت وارد کنيد | **تعداد افراد ................................**  88. نمی دانم/يادم نيست  99. بدونپاسخ |  |
| 1303 | آيا در مطالعه مشابه "مطالعه سرولوژيکی و رفتاری HIV در زنان آسيب پذير" که در سال 1388 انجام شد، شرکت کرده ايد؟ | 1. بلی  2. خير  88. نمی دانم/يادم نيست  99. بدون پاسخ |  |
| 1304 | آیا در شش ماه گذشته، يک گردنبندیادگاری دریافت کرده اید؟ | 1. بلی  2. خير  88. نمی دانم/يادم نيست  99. بدون پاسخ | 2**🡨1314** |
| 1305 | آیا می توانید گردنبند را به من نشان دهید؟ | **1.** بلی  **2. همراهم نیست**  **99.** بدون پاسخ | 1**🡨1307** |
| 1306 | آيا گردنبند را به کس ديگری داديد؟ | **1.** بلی  **2. خير**  **99.** بدون پاسخ |  |
| 1307 | آیا می توانید برای من توصیفش کنید؟ (میشه بگین چه شکلیه؟) | **1. توصیف مصاحبه شونده صحیح بود.**  **2. توصیف مصاحبه شونده نادرست بود.**  **99.** بدون پاسخ |  |
| 1308 | نوع گردنبند را مشخص کنيد؟با توجه به توصيفهای فرد، نوع گردنبند را مشخص کنيد | **1. گردنبند نگين دار بنفش**  **2. گردنبند نگين دار سبز**  **3. گردنبند نگين دار سرمه ای**  **4. گردنبند نگين دار قرمز**  **5. گردنبند سه حلقه**  **99. بدون پاسخ** |  |
| 1309 | آیا این اون گردنبندی هست که دریافت کردید؟ (آیا این شکلیه؟) لطفا گردنبند را به او نشان دهید. | 1. بلی  2. خير  99. بدون پاسخ |  |
| 1310 | چندتا از این گردنبندها را دریافت کرده اید؟ | **...................................عدد** |  |
| 1311 | آیا به خاطر دارید که چند وقت پیش این گردنبند را دریافت کردید؟ اگر به خاطر نمی آورد، لطفا بهترین حدسش را ثبت کنید. | | هفته | ماه | | | --- | --- | --- | |  |  |  |   88. نمی دانم/يادم نيست  99. بدون پاسخ |  |
| 1312 | آیا به خاطر دارید که کجا این گردنبند را دریافت کردید؟ | **..................................................** |  |
| 1313 | آیا به خاطر دارید که چه کسی این گردنبند را به شما داد؟ گزينه ها را نخوانيد و اجازه دهيد خودش بگويد | **1. دوست**  **2. شریک جنسی**  **3. مدد کار اجتماعی**  **4. يک نفر از تيم سيار که شال قرمز داشت**  **5. همکار**  **6. غریبه**  **7. سایر................................**  88. نمی دانم/يادم نيست  99. بدونپاسخ |  |
| 1314 | آیا در يکسال اخير به مرکزپزشکان بدون مرز(دروازه غار) برای دريافت خدمات مراجعه کرده ايد؟ | 1. بلی  2. خير  88. نمی دانم/يادم نيست  99. بدون پاسخ |  |
| 1315 | آیا در يکسال اخير از مرکز مرکز مشاوره ویژه زنان آسیب پذیر ولی عصر خدمت "مشاوره و انجام تست اچ آی وی" دریافت کرده اید؟ | 1. بلی  2. خير  88. نمی دانم/يادم نيست  99. بدون پاسخ |  |
| 1316 | آیا در يکسال اخير از مرکز جوانان خدمت "مشاوره و تست اچ آی وی" دریافت کرده اید؟ | 1. بلی  2. خير  88. نمی دانم/يادم نيست  99. بدون پاسخ |  |
| 1317 | آیا در یک سال اخیر در مرکز آهنگ رهایی شرق خدمات دريافت کرده ايد؟ | 1. بلی  2. خير  88. نمی دانم/يادم نيست  99. بدون پاسخ |  |
