# Supplementary material for: Population size estimation of female sex workers in Iran: Synthesis of methods and results
Source: PLoS One. 2017 Aug 10;12(8):e0182755. doi: 10.1371/journal.pone.0182755 (PMC5552099; doi:10.1371/journal.pone.0182755)
Supplement: S4 File — (DOC) [file pone.0182755.s005.doc]

This questionnaire is a part of a large questionnaire with different questions and included in an integrated Bio-Behavioral Survey (IBBS). We had different questions for different cities. Here we present the questionnaire for Tehran city. We estimated the number of FSWs for Wisdom of the Crowd (WOTC) and Multiplier.

| Number | Question | Response | Refer |
| --- | --- | --- | --- |
| 1301 | In your opinion, how many women are in this city that have sexual contacts with men for money, drugs or any other service (food, telephone card, a place for living, traveling ticket or…) | -How many do you imagine? …..  -The minimum number do you imagine? …....  -The maximum number do you imagine? ……..  88. I don’t know/I cannot remember  99. without response | 0 person  **🡪**  **1303** |
| 1302 | How many of them are over 18  If they reported the percentage, please calculate the number and write it here | -The number …..  88. I don’t know/I cannot remember  99. without response |  |
| 1303 | Did you contribute in a similar survey “Biobehavioral survey for HIV in FSWs” that conducted in 1388? | 1.Yes  2.No  88. I don’t know/I cannot remember  99. without response |  |
| 1304 | Did you receive a necklace in last 6 months? | 1.Yes  2.No  88. I don’t know/I cannot remember  99. without response | No  **🡪**  **1314** |
| 1305 | Could you show me the necklace? | 1.Yes  2.It is not with me  99. without response |  |
| 1306 | Did you pass the necklace to any else? | 1.Yes  2.No  99. without response |  |
| 1307 | Could you explain it? | 1.The explain is correct  2.The explain isn’t correct  99. without response |  |
| 1308 | Define the necklace | 1.A purple jeweled necklace  2.A green jeweled necklace  3.A navy blue jeweled necklace  4. A red jeweled necklace  5. A three-ring necklace  99. without response |  |
| 1309 | Did the necklace is similar to this? | 1.Yes  2.No  99. without response |  |
| 1310 | How many necklaces did you get? | ……………..number  88. I don’t know/I cannot remember  99. without response |  |
| 1311 | Do you remember when did you receive the necklace?  If she cannot remember, please write down the best imagination. | Month………, Week…..  88. I don’t know/I cannot remember  99. without response |  |
| 1312 | Do you remember where did you receive the necklace? | ……………….. |  |
| 1313 | Do you remember who passed you the necklace?  Please do not read the choices and let her to tell. | 1.A friend  2.Client/partner  3.Social worker  4.A person with red scarf with outreach team  5.Co-worker  6. A strange  7.Others  88. I don’t know/I cannot remember  99. without response |  |
| 1314 | Have you ever receive any service of “Darvazeh Ghar center”? | 1.Yes  2.No  88. I don’t know/I cannot remember  99. without response |  |
| 1315 | Have you ever receive counseling and testing service from “ValiAsr center”? | 1.Yes  2.No  88. I don’t know/I cannot remember  99. without response |  |
| 1316 | Have you ever receive counseling and testing service from “Javanan center”? | 1.Yes  2.No  88. I don’t know/I cannot remember  99. without response |  |
| 1317 | Have you ever receive any service from “Ahang Rahaee Shargh center”? | 1.Yes  2.No  88. I don’t know/I cannot remember  99. without response |  |
